# Perioperative Management of Severe Traumatic Brain Injury: What Is New?

**DOI:** 10.1007/s40140-018-0286-1

**Published:** 2018-08-14

**Authors:** Deacon Farrell, Audrée A. Bendo

**Affiliations:** Downstate Medical Center, State University of New York (SUNY), 450 Clarkson Avenue, Box 6, Brooklyn, New York 11203 USA

**Keywords:** Guidelines, Traumatic brain injury, Primary injury, Secondary injury, Inflammation, Apoptosis, Excitotoxicity, Neuroprotection, Dexmedetomidine, Ketamine, Cannabinoids, Progesterone, Amantadine, Rehabilitation

## Abstract

**Purpose of the Review:**

Severe traumatic brain injury (TBI) continues to represent a global public health issue, and mortality and morbidity in TBI patients remain substantial. There are ongoing international collaborations to provide guidelines for perioperative care and management of severe TBI patients. In addition, new pharmacologic agents are being tested along with cognitive rehabilitation to improve functional independence and outcome in TBI patients. This review will discuss the current updates in the guidelines for the perioperative management of TBI patients and describe potential new therapies to improve functional outcomes.

**Recent Findings:**

In the most recent guidelines published by The Brain Trauma Foundation, therapeutic options were reviewed based on new and revised evidence or lack of evidence. For example, changes and/or updates were made to the recommendations for the use of sedation and hypothermia in TBI patients, and new evidence was provided for the use of cerebrospinal fluid drainage as a first-line treatment for increased intracranial pressure (ICP). In addition to the guidelines, new ‘multi-potential’ agents that can target several mechanisms are being tested along with cognitive rehabilitation.

**Summary:**

The major goal of perioperative management of TBI patients is to prevent secondary damage. Therapeutic measures based on established guidelines and recommendations must be instituted promptly throughout the perioperative course to reduce morbidity and mortality.

## Introduction

Traumatic brain injury (TBI) is a major cause of death and disability with large direct and indirect costs to society. It affects roughly 3.3–5.3 million people per year in the USA, and the annual direct cost of TBI has been estimated to be $9.2 billion per year ($13.1 billion in 2013) [1, 2]. An additional $51.2 billion is lost through missed work and lost productivity [3]. The leading causes of TBIs are falls, motor vehicle crashes, and assaults with males twice as likely to be affected as females [3]. Traumatic brain injury (TBI) is also a significant problem in older adults. In persons aged 65 and older, TBI has been to be responsible for more than 80,000 emergency department visits each year, approximately three-quarters of which result in hospitalization [4•]. Adults aged 75 and older have the highest rates of TBI-related hospitalization and death. Falls are the leading cause of TBI for older adults (51%), and motor vehicle traffic crashes are second [4•].

In 1995, the Brain Trauma Foundation approved guidelines for the initial resuscitation of severely head-injured patients and treatment of intracranial hypertension recognizing the need to standardize care to improve outcomes [5]. These guidelines represent a comprehensive review of the literature and provide the best treatment recommendations for the acute care management of the hospitalized TBI patient. Resuscitation protocols from pre-hospital to critical care management have been developed and instituted based on current literature and evidence. The 4th edition of the Brain Trauma Foundation guidelines were published in 2016. This review will focus on recent findings including the updated guideline recommendations and novel treatments including new therapeutic agents and rehabilitation with the potential to prevent secondary brain injury and improve patient morbidity after TBI.

### Classification of Severe TBI and Pathophysiology

Classification of TBI has been traditionally based on the Glasgow Coma Scale (GCS) which defines neurologic impairment in terms of eye opening, speech, and motor function. The total score is 15 and severe head injury is determined by a score of 8 or less. In general, mortality is closely related to the initial score on the GCS [6].

**Fig. 1:**
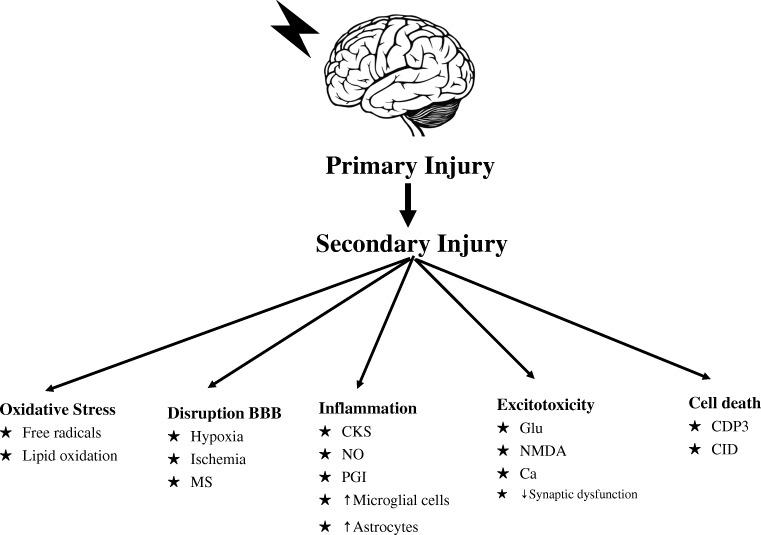
Secondary injury from oxidative stress, disruption of the blood-brain-barrier (BBB), inflammation, excitotoxicity, and cell death and resulting factors involved in neuronal damage. MS: mitochondrial stress, CKS: cytokines, NO: nitric oxide, PGI: prostaglandins, Glue: glutamate, NMDA: *N*-methyl-D-aspartate receptor, Ca: calcium, CDP: caspase-dependent 3, CID: caspase-independent factor

Following TBI, the primary injury results from the mechanical effect of forces applied to the skull and brain at the time of the insult. The primary injury causes damage to neuronal tissue which initiates an endogenous neuroinflammatory response contributing to the development of blood-brain barrier breakdown, cerebral edema, further increases in intracranial pressure (ICP), and ultimately, cell death by apoptosis and necrosis, if untreated. Secondary injury begins immediately after primary injury and continues to evolve for extended periods of time causing global and focal ischemia, worsening survival, and morbidity (Fig. 1) [6]. The secondary injury cascade may be modified to improve outcomes.

The general principles of early management are to maintain adequate oxygenation, stable cerebral perfusion, glycemic control, and maintenance of electrolytes. The clinical management goal in TBI patients is to initiate timely and appropriate therapy to prevent secondary brain injury.

### Brain Trauma Foundation Guidelines

The current 4th edition of The Brain Trauma Foundation Guidelines for the Management of Severe TBI can be found at http://www.braintrauma.org/coma/guidelines. Recent updates in the guidelines reflect the most current evidence. The primary focuses of the 4th edition were to integrate TBI-specific, evidence-based recommendations with clinical best practices for trauma patients and to provide guidelines or suggestions where evidence is insufficient. Details on the changes within each topic are listed in the latest edition of the guidelines and are reported in the sections for each topic in the guideline document.

The literature analyzed in the 4th edition by *Carney* et al. [8••] used GCS to classify TBI and assessed outcomes in mortality, morbidity, and neurological function where they were compared [8••]. Recommendations in the guidelines were assigned into categories Level I, Level-II A, Level II-B, Level III depending on the assessment of the quality of evidence [8••]

The following discussion includes recommendations from the most recent edition of the guidelines published by the braintraumafoundation.org.

1. Decompressive craniectomy to reduce ICP. It can be performed unilaterally or bilaterally and can be approached via temporal, frontal, or circumferential excisions. Due to the variety of methods used by neurosurgeons for this procedure, the strength of research on results is lacking. The DECRA (Decompressive Craniectomy in Patients with Severe Traumatic Brain Injury) trial compared decompressive craniotomy to medical therapy in reducing ICP and found that decompressive craniotomy was associated with worse functional outcomes than compared to medical care [9].

### Updated Treatment Recommendations:

#### Level IIA

Bifrontal decompressive craniectomy is not recommended to improve outcomes as measured by the Glasgow Outcome Score (GOS) at 6 months post-injury in severe TBI patients with diffuse injury (without mass lesions) and with ICP elevation to values > 20 mmHg for more than 15 min within a 1-h period that are refractory to first tier therapies [10]. However, this procedure has been demonstrated to reduce ICP and to minimize days in the ICU. A large frontotemporoparietal decompressive craniectomy (not less than 12 × 15 or 15 cm diameter) is recommended over a small frontotemporoparietal craniectomy for reduced mortality and improved neurologic outcomes in patients with severe TBI [10].

2. Hyperosmolar agents are used in the management of ICP. Mannitol therapy is often immediately initiated in patients suspected of intracranial hypertension with impending signs of herniation. However, a systematic Cochrane review found that there was “insufficient reliable evidence to make recommendations for the use of mannitol in the management of patients with TBI” [7, 11]. Hyperosmolar therapy is assumed to be beneficial on the basis of its ability to lower ICP, but no trials have been carried out in which hyperosmolar therapy has been omitted from the treatment regimen. Hence, the current level of evidence is insufficient to support the use of a specific hyperosmolar therapy for improving clinical outcomes. The Level II and III recommendations from the *3rd Edition* of the Brain Trauma Foundation guidelines were not carried forward because they were derived from studies that did not meet criteria for inclusion. However, the Brain Trauma Foundation guideline committee included the *3rd Edition* recommendations on this topic in the *4th edition* in recognition of the need for hyperosmolar therapy to reduce ICP, while acknowledging the need for more research to make evidence-based recommendations.

### Updated Treatment Recommendations:

Although hyperosmolar therapy may lower ICP, there was insufficient evidence about effects on clinical outcomes to support a specific recommendation or to support use of any specific hyperosmolar agent, for patients with severe TBI.

3. Sedation is used in the management of patients with TBI. Barbiturates and propofol are variably used with the goal of reducing elevated ICP and terminating seizure activity [12]. Barbiturates stimulate γ-aminobutyric acid (GABA) receptors and inhibit α-amino-3-hydroxy-5-methyl-4-isoxazolepropionic acid (AMPA) receptors in the CNS producing dose-dependent sedation and general anesthesia. Thiopental has a high lipid solubility which allows for rapid transfer across the blood-brain barrier and fast onset of action. Barbiturates also have been studied prospectively and found to decrease ICP and flow velocity in the middle cerebral artery [12, 13]. Thiopental has been shown to have beneficial effects on cerebral blood flow (CBF) and cerebral metabolic rate (CMRO_2_). However, it is important to note that thiopental can cause hypotension which can offset the beneficial effects of lowering ICP by decreasing cerebral perfusion pressure (CPP) [14].

### Updated Treatment Recommendations:

#### Level IIB

Administration of barbiturates to induce burst suppression measured by EEG as prophylaxis against the development of intracranial hypertension is not recommended in the current guidelines. The use of high-dose barbiturate administration is recommended only to control elevated ICP that fails to respond to standard medical and surgical therapy. It is important to maintain hemodynamic stability during barbiturate therapy as the patient may become hypotensive.

Propofol, a lipid soluble agent that acts on gamma-aminobutyric acid receptor A (GABA*a*), has been shown to decrease ICP and CBF and CMRO_2_ [15, 16]. However, a reduction in mean arterial blood pressure (MAP) may reduce the CPP, if this is not mitigated with adequate fluid resuscitation and vasopressors [15]. Propofol use for sedation in patients with TBI can be used for the control of ICP, but has failed to show improvement in mortality for 6-month outcomes [16]. Caution is required as high-dose propofol can produce propofol infusion syndrome which can cause significant morbidity.

### Updated Treatment Recommendation:

#### Level IIB

Propofol sedation is recommended for the control of ICP, but has failed to show improvement in mortality for 6-month outcomes [15].

### Additional Sedative Agents:

The following agents have been shown to have potential role in the management of TBI patients**,** although they are not included in the current Brain Trauma Foundation guidelines.

- Dexmedetomidine, a highly selective α2-adrenergic agonist, is an intravenous sedative mainly used in the ICU setting. Dexmedetomidine induces sedation by decreasing activity of noradrenergic neurons in the locus ceruleus, thereby increasing the activity of inhibitory gamma-aminobutyric acid neurons in the ventrolateral preoptic nucleus [17]. Dexmedetomidine has favorable effects on heart rate, blood pressure, and agitation making a useful sedation agent in vented TBI patients. *Lump* et al. showed that it may be effective for the management of paroxysmal autonomic instability with dystonia which can present in patients with TBI [18]. Other studies have shown that infusion of dexmedetomidine in TBI patients can lead to a decrease use of narcotics and sedative [19]. Dexmedetomidine has also been shown to have a role in treating refractory hypertension while reducing the amount of mannitol used in ICU patients [20].
- Ketamine, an N-methyl-D-aspartate receptor antagonist, is gaining acceptance for induction, maintenance, and sedation in patients with TBI. Ketamine was traditionally avoided in the management of patients with TBI due to concerns that it increased ICP. Recent evidence has suggested the potential benefits of ketamine. A systematic review demonstrated that ICP did not increase in any of the studies during ketamine administration, and there were no significant adverse events reported related to ketamine administration [21••]. The antagonism of NMDA receptors by ketamine can decrease the release of neurotoxic glutamate and may impart a protective effect in patients with traumatic brain injury [22, 23]. New studies are investigating the role of ketamine in brain injury. A recent clinical study looked retrospectively at the effect of ketamine on the incidence of spreading depolarization in a continuum of neurologic disease including TBI, SAH, and malignant stroke and found a consistent inhibitory effect on neuronal discharges across all injury modalities [24]. Spreading depolarization has been shown to worsen flow-metabolism coupling and excitotoxicity, and therefore, ketamine may have these effects and provide neuroprotection in this context.

4. Ventilation therapies is a title change from “Hyperventilation” which was discussed in previous editions [8••]. The reason for the title change is to include related therapies in future guidelines. Hyperventilation is administered to patients with TBI for ICP control and reversing brain and cerebrospinal fluid (CSF) acidosis. Possible disadvantages include cerebral vasoconstriction to such an extent that cerebral ischemia ensues [25]. Therefore, close monitoring of PaCO_2_ is recommended with avoidance of both hypocapnia and hypercapnia which can be harmful. The recommendation for hyperventilation in the previous edition of guidelines was not carried forward in the 4th edition. Moreover, there was insufficient evidence to support a Level I or II A recommendation for this topic.

### Updated Treatment Recommendations:

#### Level IIB

Prolonged prophylactic hyperventilation with PaCO_2_ of ≤ 25 mmHg is not recommended.

Note:

The Level III recommendations from the 3rd Edition of these guidelines were not carried forward because they were derived from case series studies. While no evidence is available from comparative studies to support a formal recommendation, the Brain Trauma Foundation Committee chose to re-state here the 3rd Edition Level III recommendations. Their rationale for doing so is to maintain sufficient recognition of the potential need for hyperventilation as a temporizing measure. See below.

Recommendations from the prior 3rd Edition guideline

- Hyperventilation is recommended as a temporizing measure for the reduction of elevated ICP.
- Hyperventilation should be avoided during the first 24 h after injury when CBF is often critically reduced.
- If hyperventilation is used, jugular venous oxygen saturation (SjO2) or brain tissue O2 partial pressure (BtpO2) measurements are recommended to monitor oxygen delivery.

5. Cerebrospinal fluid drainage is a treatment that can be used to manage severely elevated ICP. An external ventricular drain (EVD) is often placed to measure ICP in patients with TBI. There is an increased interest into the potential added benefit of cerebrospinal fluid (CSF) drainage in which recent studies have shown improved outcomes in patients with TBI. There is no consensus regarding the optimal method of cerebrospinal fluid removal [26].

### Updated Treatment Recommendations:

#### Level III

An EVD system zeroed at the midbrain with continuous drainage of CSF may be considered to lower ICP burden more effectively than intermittent use [27••]. The use of CSF drainage to lower ICP in patients with an initial GCS < 6 during the first 12 h after injury may be considered.

6. Hypothermia. Over the years, there has been considerable research interest in hypothermia as a means to reduce tissue damage associated with central nervous system trauma. However, there is no evidence that benefit is obtained with hypothermia therapy. Hypothermia treatment is described as either “prophylactic” or “therapeutic”. When it is administered early after injury and prior to ICP elevation, it is termed “prophylactic”. When it is administered as a treatment for refractory ICP elevation, it is termed “therapeutic”. Most studies on “prophylactic hypothermia” report conflicting results [28]. However, hypothermia continues to be administered as a third-tier therapy in patients with refractory intracranial hypertension. The use of hypothermia is associated with an increased incidence of adverse events (e.g., coagulopathy, immunosuppression, and cardiac dysrhythmia) and a lack of improvement in outcome compared to normothermic patients. When applying the new standards for study inclusion in the *4th Edition* of the Guidelines, the authors could not support the previous recommendations made in the *3rd Edition* for studies comparing hypothermia with normothermia. The study treatments were considered clinically different and not appropriate for meta-analysis [29]. Therefore, the report concluded that there was insufficient evidence to support a Level I or IIA recommendation for this topic [8••].

### Updated Treatment Recommendations:

#### Level IIB

The use of prophylactic hypothermia, early, within 2.5 h and short-term, 48 h post-injury, is not recommended to improve outcomes in patients with diffuse injury.

7. Deep vein thrombosis. Low-molecular-weight heparin (LMWH) is a class of anticoagulation medication that is used to treat deep vein thrombosis and reduce the risk of developing pulmonary embolism in bed bound patients. There has been considerable interest in the safety profile for LMWH in patients with TBI. Patients not on anticoagulation are at increased risk of developing deep vein thrombosis.

### Updated Treatment Recommendations:

#### Level III

Low-dose unfractionated heparin or LMWH may be used in combination with mechanical prophylaxis once the intracranial hemorrhage is stable [30••]. There is a potential risk for expansion of intracranial hemorrhage so the appropriate time to initiate anticoagulation would be based on clinical guidance. There is insufficient evidence to support recommendations regarding the preferred agent, dose, or timing of pharmacologic prophylaxis for deep vein thrombosis [31]. Other methods, such as compression stocking and maintaining normovolemia, should be implemented to prevent deep vein thrombosis.

8. Infection prophylaxis. Infection risk is considered to be high in TBI patients. Respiratory tract infections are the most common among TBI patients, with a notable predominance of Acinetobacter reported as a ventilator-associated pneumonia (VAP) pathogen [32••].

### Updated Treatment Recommendations:

#### Level IIA

Tracheostomy is recommended to reduce mechanical ventilation days to avoid ventilator deconditioning in patients when the overall benefit outweighs the complications associated with performing a tracheostomy. Tracheostomy was also considered a potential way of reducing VAP. However, there is no evidence that early tracheostomy reduces mortality or the rate of nosocomial pneumonia. The use of povidone-iodine in oral care is not recommended to reduce VAP. PI oral care has been associated with an increased risk of acute respiratory distress syndrome [33].

#### Level III

It has also been noted that Antimicrobial-impregnated catheters may be considered to prevent catheter-related infections during external ventricular drainage [32••].

9. Nutrition. TBI results in a hypermetabolic state that increases systemic and cerebral energy requirements. Achieving adequate nutrition to meet this demand has been difficult to define in TBI patients. A study by Hartle and colleagues found that patients who were not fed within the first week after TBI had significant increases in mortality. Early enteral nutrition (EN) also showed benefit compared with more delayed traditional EN in terms of infections and overall complications and longer term outcomes 3 months post-injury [34]. The issue of nutrition leads directly into glycemic control as it has long been known that an increase in serum glucose is observed after severe stress, including severe TBI [35]. Studies from other critical illnesses have demonstrated that controlling this response with the use of insulin can lead to significant improvements in outcomes of critically ill patients [36]. However, a similar approach in a population of adults with severe TBI demonstrated a worrisome pattern of metabolic responses within the brain interstitial fluid, implying that the practice of “tight glucose control” could have deleterious effects in patients with severe TBI [37]. The Brain Trauma Foundation guidelines have attempted to address these recent studies where evidence is strong, but does not address other questions like glycemic control underscoring the need for more research on nutrition in TBI patients. There was insufficient evidence to support a Level I recommendation for this topic

### Updated Treatment Recommendation

#### Level IIA

Feeding patients to attain basal caloric replacement at least by the fifth day and, at most, by the seventh day post-injury is recommended to decrease mortality.

#### Level IIB

Transgastric jejunal feeding is recommended to reduce the incidence of ventilator-associated pneumonia.

10. Seizure prophylaxis. In patients with severe TBI, the rate of clinical post-traumatic seizures (PTS) may be as high as 12%, while that of subclinical seizures detected on electroencephalography may be as high as 20 to 25% [38]. PTS are classified into early when < 7 days or late when they occur after 7 days. Seizures often occur as result of hematoma formation, presence of retained foreign body, depressed skull fractures, GCS score less than 10, and amnesia [39]. There is little evidence regarding the appropriate administration of anti-seizure medication in TBI patients. The Brain Trauma Foundation guidelines set out to clarify the evidence for the use of phenytoin and other anti-seizure medications. There was insufficient evidence to support a Level I recommendation for this topic

### Updated Treatment Recommendation

#### Level IIA

Prophylactic use of phenytoin or valproate is not recommended for preventing late PTS. However, phenytoin is recommended to decrease the incidence of early PTS (within 7 days of injury), when the overall benefit is thought to outweigh the complications associated with such treatment. However, early PTS have not been associated with worse outcomes. There is insufficient evidence for the use of levetiracetam over phenytoin in preventing early PTS and toxicity.

11. Steroids. The beneficial effects of steroid therapy in brain tumor patients have not been demonstrated in patients with TBI. It was thought that patients with elevated ICP and brain edema may benefit from corticosteroids by reducing mortality. Although steroid delivery results in decreased cerebrospinal fluid production, restores homeostatic vascular permeability, and decreases edema, these effects have not translated to a decrease in ICP and reduction in morbidity and mortality rates [40]. The studies examined by the Brain Trauma Foundation committee found that out of the five studies reviewed, patients did not benefit from steroid treatment. One of the studies showed that methylprednisolone increased mortality causing the trial to be stopped [41–45]. There were no changes made to the recommendations from the prior *3rd Edition* regarding steroid administration in TBI patients.

### Updated Treatment Recommendation

#### Level I

The use of steroids is not recommended for improving outcome or reducing ICP. In patients with severe TBI, high-dose methylprednisolone was associated with increased mortality and is contraindicated.

### Brain Trauma Foundation Monitoring Guidelines

The goal of the medical management of severe TBI is to ensure that nutrient delivery to the brain is optimized through the period of abnormal physiology and brain swelling that follows the injury. Treatment informed by data from monitoring may result in better outcomes than treatment informed solely by data from clinical assessment [8••]. These recommendations are related to the influence on patient outcomes of three types of monitoring: ICP, cerebral perfusion pressure (CPP) monitoring, and advanced cerebral monitoring. It is important to note that prior editions addressed several questions in this section. The topic of monitoring is now focused on whether monitoring results in better outcomes.

### Updated Monitoring Recommendations

- Intracranial pressure monitoring

#### Level IIB

Management of severe TBI patients using information from ICP monitoring is recommended to reduce in-hospital and 2 weeks post-injury mortality.

Additionally, while no evidence was available from comparative studies to support a formal recommendation, the Brain Trauma Foundation chose to re-state the 3rd Edition recommendations for ICP monitoring in patients with severe TBI and abnormal CT scan. According to the 3rd edition, ICP monitoring was also indicated in patients with severe TBI with a normal CT scan when more than ≥ 2 of the following features are noted at admission: age > 40 years, unilateral or bilateral motor posturing, or systolic blood pressure < 90 mmHg.

- Cerebral perfusion pressure monitoring

CPP is the difference between the mean arterial blood pressure and ICP. Views on the optimal CPP have evolved over the years. It has been suggested that an optimal CPP value may need to be tailored to individual patients and that achieving this level throughout the course of a patient’s care could be associated with better outcomes [3].

#### Level IIB

Management of severe TBI patients using guidelines-based recommendations for CPP monitoring is recommended to decrease 2-week mortality.

- Advanced cerebral monitoring

These monitors include Brain Tissue Oxygen Monitoring (PbtO_2_) and Jugular Bulb Monitoring of Arteriovenous Oxygen Content Difference (AVDO_2_).

#### Level III

Jugular bulb monitoring of AVDO_2_ may be considered to provide management decisions in TBI patients. Jugular venous saturation < 50% may be a threshold to avoid in order to reduce mortality and improve 3- and 6-month outcomes.

### Threshold Recommendations

In the *4th Edition*, thresholds for blood pressure, ICP, cerebral perfusion pressure, and advanced cerebral monitoring are included. These threshold values from the guidelines are used to create parameters for in-hospital patients and guide management of severe TBI. These thresholds can be a value to avoid in order to decrease the probability of negative outcomes or a value to aim for in order to increase the probability of positive outcomes, and it can be a value that triggers a change in treatment.

- Blood pressure thresholds

#### Level III

Maintaining SBP at ≥ 100 mmHg for patients 50–69 years old or at ≥ 110 mmHg or above for patients 15–49 or 70 years old may be considered to decrease mortality and improve outcomes.

- ICP thresholds

#### Level IIB

Treating ICP > 22 mmHg is recommended because values above this level are associated with increased mortality.

#### Level III

A combination of ICP values and clinical and brain CT findings may be used to make management decisions.

- Cerebral perfusion pressure thresholds

#### Level IIB

The recommended target of CPP value for survival and improved outcomes is between 60 and 70 mmHg. Currently, it is unclear whether 60 or 70 mmHg is the minimum optimal CPP threshold and may depend on patient autoregulatory status.

#### Level III

Avoid having CPP > 70 mmHg with fluids and vasopressors due to increased risk of respiratory failure

### Potential New Therapies

Research published over several decades have sought to elucidate mechanisms of secondary injury with the intention of developing neuroprotective treatments. The importance of viewing injury more broadly to include endothelial cells, astroglia, microglia, oligodendroglia which are viewed as neuronal support structures and involved in the regeneration of neuronal tissue. Preclinical studies have suggested many promising pharmacological agents which are in phase III prospective clinical trials. The potential therapy direction is now to focus research efforts on multi-targeted pharmacological agents in early intervention to reduce the cascade of secondary injury. Some examples of multi-potential drugs and their targets are listed in Table 1 which describes different types of injury that are involved in neuronal damage in TBI [46••].

**Table 1:** Potential pharmacological agents that target an array of signaling pathways known to be involved in neuronal injury that are currently under investigation in patients with TBI [30]

Pharmacological agents	Secondary injury targets
Amantadine	Excitotoxicity, BBB
Progesterone	Oxidative stress, apoptosis, inflammation
Cannabinoids	Inflammation, excitotoxicity, oxidative stress

*BBB* blood-brain barrier

The importance of viewing injury more broadly to include endothelial cells, astroglia, microglia, oligodendroglia which are viewed as neuronal support structures and involved in the regeneration of neuronal tissue. Perhaps the most important recent observations relate to the potential role of apoptosis and necrosis in secondary brain injury and disruption of these support structures. The following pharmacological agents are currently being clinically investigated to determine whether they disrupt secondary injury cascades and provide improved clinical outcomes [47].

Amantadine appears to act as an N-methyl-D-aspartate antagonist and indirect dopamine agonist [48]. Amantadine is one of the most commonly prescribed medications for patients with prolonged disorders of consciousness after traumatic brain injury. Preliminary studies have suggested that amantadine accelerated the pace of functional recovery during active treatment in patients with post-traumatic disorders of consciousness [46••]. Exposure of amantadine is associated with a more rapid emergence of cognitively mediated behaviors that are involved in regaining functional independence [49].

Progesterone has been shown to have broad neuroprotective properties which include inhibition of inflammation cytokines, reduction of inflammation factors, prevention of BBB disruption, and control of vasogenic edema [50]. In addition, progesterone has been shown to prevent excitotoxicity and limit apoptosis by preventing biochemical insults, such as calcium (Ca2+) flux and nitric oxide production and by decreasing levels of caspase 3, a known molecule in the apotosis pathway [51•].

Two-phase 2 randomized controlled trials with progesterone have shown clinical benefit. Preliminary clinical data obtained with the use of various progesterone formulations and routes of delivery, combined with experimental data showing adequate brain penetration, provided initial support for a neuroprotective role of progesterone in TBI [52]. The PROTECT trial involved 100 patients within 11 h after injury, with a 72-h treatment duration showed an association with a reduction in the rate of death from any cause, as compared with placebo. Another trial using progesterone treatment, which was initiated within 8 h after injury by means of intramuscular injection, with a120-h treatment duration, was associated with reduced mortality, as compared with placebo [52].

Cannabinoids bind to Cannabinoid receptors for which there are two types, Cannabinoid type 1 (CB1) and Cannabinoid type 2 (CB2). They are found in the endocannabinoid system of central nervous system (CNS) [53]. The neuroprotective effects of cannabinoids include inhibition of the release of glutamate and inflammatory cytokines [53–56]. Since the legalization of medicinal cannabinoid compounds, multicenter, randomized controlled trials involving TBI patients are underway.

Currently, there is one trial investigating a synthetic, non psychotropic cannabinoid, HU-211 (dexanabinol). This compound was found to exhibit pharmacological properties characteristic of a noncompetitive NMDA-receptor antagonist [57, 58]. HU-211 also blocks tumor-necrosis factor synthesis and has antioxidant properties, inhibiting release of ROS. Because glutamate, ROS, and tumor-necrosis factor are well known to be involved in the pathophysiology of brain injury [59], the above observations have led to clinical trials. Phase I and II trials have demonstrated [53] that HU-211 significantly improves the neurological outcome of patients with TBI.

### Role of Rehabilitation

Cognitive impairments due to TBI are substantial sources of morbidity for affected individuals, their family members, and society. Cognitive testing has become a method used to assess performance and various functions in rehabilitating patients with TBI. The areas assessed include attention, memory, learning, mental organization, affect, and expression with executive functions. A detailed neuropsychiatric assessment to determine existing cognitive abilities and inabilities of the TBI patient is required before starting cognitive rehabilitation. In addition, repeat neuropsychological assessments, at a regular interval, are necessary to evaluate the effectiveness of ongoing treatment [60].

Cognitive rehabilitation should not be used as a “stand alone” therapy for patients with cognitive deficits. It has been shown to be more effective when implemented as part of a multidisciplinary/interdisciplinary approach [61•]. The multidisciplinary team approach for effective cognitive rehabilitation would require the involvement of physicians, neuropsychologists, speech-language pathologists, occupational therapists, physical therapist, and social workers. Timing for initiation of cognitive rehabilitation has been under reported in the literature. *Andelic* et al. demonstrated that rehabilitation which was initiated earlier in TBI patients had higher Glasgow Outome Scale Extended (GOSE) and Disability Rating Scale (DRS) compared to patients who had a later intervention [62].

### Computer Technology

The use of virtual reality (VR) technology which involves audio and visual stimulations that engage different components of impairment such as memory, attention, and visual perception greatly improves patients’ participation in training and rehabilitation. The use of computer technology has been shown to have advantages for assessment and training of cognitive impairment compared with cognitive training by rehabilitation therapists [63].

### Brain Stimulation Techniques

Repetitive transcranial magnetic stimulation (RTMS) is a painless, noninvasive, easily operated treatment with few adverse reactions. *Neville* et al. showed RTMS improved cognitive function in patients with TBI [64]. Several studies have shown that RTMS reduce TBI-associated depression, tinnitus, neglect, memory deficits, and attention disorders [65].

### Behavioral Emotional Therapy

Several studies have shown that post-traumatic outcomes can be influenced by psychological factors like stress, anxiety, perception of illness, symptom expectations, litigation, and/or premorbid psychiatric conditions [66••]. Post-traumatic stress disorder (PTSD) is frequently associated with mild TBI [66••]. This would indicate a possible role for early initiation of psychological intervention and pharmacotherapy to reduce the occurrence of psychiatric conditions.

### Pharmacotherapy in Rehabilitation

Pharmacotherapy with catecholaminergic and cholinergic properties has also been found to be useful adjuncts in cognitive rehabilitation [67]. Psychostimulants and other dopaminergic active agents, for example, methylphenidate, dextroamphetamine, amantadine, levodopa/carbidopa, bromocriptine, may modestly improve arousal and speed of information processing, reduce distractibility, and improve some aspects of executive function [68••].

TBI patients may need the long-term support of healthcare professionals, including cognitive rehabilitation as well as social, vocational, and family support. Indeed, recent findings have suggested that specific community re-entry services may be useful for preventing long term patient deterioration [69].

## Discussion and Conclusions

### Updated Guidelines

The Brain Trauma Foundation guidelines published in the *4th edition* examined 102 new articles which were used as evidence to update the guidelines [8••]. These can be found in Table 4 of the appendix section of the *4th edition* of the guidelines, https://www.braintrauma.org/coma/guidelines. Basic information about the studies is provided and includes study design, number of patients, and data class. More specific details, including outcomes and results, also are included in the evidence tables and narrative in this document. The authors state that there will be no 5th edition. They are moving to a “Living Guidelines Model” of continuous monitoring of the literature with rapid updates online when warranted [8••].

The updated guidelines for medical management of TBI patients were reviewed with the understanding that there is insufficient evidence for many of our current clinical practices [7, 8••]. However, this comprehensive review of the literature published in the 4th edition of the Guidelines provides a conceptual framework for research initiatives to address gaps in knowledge and study design flaws with the goal of developing evidence-based clinical practice guidelines.

There is no doubt that the major goal of perioperative management for TBI patients is to prevent secondary damage. Therapeutic measures based on established guidelines and recommendations must be instituted promptly throughout the perioperative course to reduce morbidity and mortality. The use of new therapeutic agents in combination with intensive rehabilitation efforts shows promise for reducing morbidity in this high-risk population.
